# Hepatitis B Virus Promotes Hepatocellular Carcinoma Progression Synergistically With Hepatic Stellate Cells *via* Facilitating the Expression and Secretion of ENPP2

**DOI:** 10.3389/fmolb.2021.745990

**Published:** 2021-11-05

**Authors:** Wanyu Deng, Fu Chen, Ziyu Zhou, Yipei Huang, Junlong Lin, Fapeng Zhang, Gang Xiao, Chaoqun Liu, Chao Liu, Leibo Xu

**Affiliations:** ^1^ Department of Biliary Pancreatic Surgery, Sun Yat-sen Memorial Hospital, Sun Yat-sen University, Guangzhou, China; ^2^ Guangdong Provincial Key Laboratory of Malignant Tumor Epigenetics and Gene Regulation, Sun Yat-sen Memorial Hospital, Sun Yat-sen University, Guangzhou, China; ^3^ College of Life Science, Shangrao Normal University, Shangrao, China

**Keywords:** hepatitis B virus-related hepatocellular carcinoma, prognosis, ectonucleotide pyrophosphatase-phosphodiesterase 2, hepatic stellate cells, progression

## Abstract

**Background:** Hepatitis B virus (HBV) infection is a major risk factor causing hepatocellular carcinoma (HCC) development, but the molecular mechanisms are not fully elucidated. It has been reported that virus infection induces ectonucleotide pyrophosphatase-phosphodiesterase 2 (ENPP2) expression, the latter participates in tumor progression. Therefore, the aim of the present study was to investigate whether HBV induced HCC malignancy *via* ENPP2.

**Methods:** HCC patient clinical data were collected and prognosis was analyzed. Transient transfection and stable ectopic expression of the HBV genome were established in hepatoma cell lines. Immunohistochemical staining, RT-qPCR, western blot, and ELISA assays were used to detect the expression and secretion of ENPP2. Finally, CCK-8, colony formation, and migration assays as well as a subcutaneous xenograft mouse model were used to investigate the influence of HBV infection, ENPP2 expression, and activated hepatic stellate cells (aHSCs) on HCC progression *in vitro* and *in vivo*.

**Results:** The data from cancer databases indicated that the level of ENPP2 was significant higher in HCC compared within normal liver tissues. Clinical relevance analysis using 158 HCC patients displayed that ENPP2 expression was positively correlated with poor overall survival and disease-free survival. Statistical analysis revealed that compared to HBV-negative HCC tissues, HBV-positive tissues expressed a higher level of ENPP2. *In vitro*, HBV upregulated ENPP2 expression and secretion in hepatoma cells and promoted hepatoma cell proliferation, colony formation, and migration *via* enhancement of ENPP2; downregulation of ENPP2 expression or inhibition of its function suppressed HCC progression. In addition, aHSCs strengthened hepatoma cell proliferation, migration *in vitro*, and promoted tumorigenesis synergistically with HBV *in vivo*; a loss-function assay further verified that ENPP2 is essential for HBV/aHSC-induced HCC progression.

**Conclusion:** HBV enhanced the expression and secretion of ENPP2 in hepatoma cells, combined with aHSCs to promote HCC progression *via* ENPP2.

## Introduction

Globally, hepatocellular carcinoma (HCC) is the dominant type of liver cancer, accounting for approximately 75% of the total (McGlynn et al., 2021). Although there have been improvements in treatments for HCC, the prognosis and survival rate for patients remain unsatisfying due to limited effective therapeutic options (Zhong et al., 2021). Hepatitis B virus (HBV) infection is the most prominent risk factor causing HCC occurrence and development in developing countries (Yang et al., 2019; Llovet et al., 2021). Viral infection causes the transformation of the liver to benefit hepatocyte malignancy *via* genome integration, activating oncogenes (Levrero and Zucman-Rossi, 2016), and changing the immune response process to an immunosuppressive microenvironment, especially in HCC patients (Liu et al., 2016; Wang et al., 2018; Li et al., 2020), resulting in virus persistence and reactivation (Shi and Zheng, 2020). HBV reactivation is an independent risk factor for HCC patient survival and antiviral treatment is associated with prolonging the overall survival (OS) (Liu S. et al., 2020) and risk reduction of tumor recurrence after curative treatment for HBV-related HCC patients (Huang et al., 2017; Huang et al., 2018), while the exact molecular mechanisms for HCC aggressiveness induced by HBV are still unclear.

Ectonucleotide pyrophosphatase-phosphodiesterase 2 (ENPP2) is a secreted lysophospholipase D and is largely responsible for converting extracellular lysophosphatidylcholine (LPC) into lysophosphatidic acid (LPA) (Salgado-Polo et al., 2018). ENPP2 is essential for normal development, is implicated in various physiological processes, and is also associated with pathological conditions including cancer (Nishimasu et al., 2012). For instance, inhibiting ENPP2 decreases initial breast tumor growth and subsequent lung metastatic nodules in mice (Benesch et al., 2014), ENPP2 is highly secreted from ovarian cancer stem cells (CSC) (Seo et al., 2016), and the ENPP2/LPA signaling axis is critical for maintaining CSC characteristics (Seo et al., 2016), facilitating estrogen-induced endometrial cancer cell proliferation (Zhang et al., 2018) and promoting K-ras-(G12D)-driven lung cancer pathogenesis (Magkrioti et al., 2018). Aberrant ENPP2 expression has been observed in several chronic inflammatory diseases or malignant diseases (Barbayianni et al., 2015), such as in chronic liver disease patients of different etiologies. ENPP2 in turn activates hepatic stellate cells (HSCs) and promotes HCC development (Enooku et al., 2016; Kaffe et al., 2017). Besides, in Hodgkin lymphoma, high levels of ENPP2 are strongly positivity associated with Epstein-Barr virus (EBV), EBV infection results in the induction of ENPP2 and leads to the enhanced growth and survival of Hodgkin lymphoma cells *via* the ENPP2/LPA axis (Baumforth et al., 2005). During hepatitis C virus infection, the plasma level of ENPP2 and LPA in patients are elevated (Kostadinova et al., 2016). It has been reported that increases in serum ENPP2 activity and protein levels have been found in patients with chronic hepatitis B (Joshita et al., 2018). This means virus infection may enhance ENPP2 expression. However, whether HBV infection regulates the expression of ENPP2 in hepatocytes and the role of ENPP2 in HBV-related HCC malignancy are still unknown. Herein, we want to verify whether ENPP2 is a factor that connects HBV infection and tumor progression in HBV-related HCC.

In the present study, we firstly analyzed the expression differences of ENPP2 between normal liver and HCC tissue, especially comparing the differences in HBV-positive and -negative HCC tissues. The prognostic correlations of ENPP2 expression and HCC patients were analyzed. We then designed a series of *in vitro* and *in vivo* studies to observe whether HBV induced HCC progression *via* enhancement of the expression and secretion of ENPP2. Finally, since in liver tissue HBV infection not only transforms host cell hepatocytes and activates HSC through viral antigens (Martin-Vilchez et al., 2008; Zan et al., 2013; Gong et al., 2016; Zhang et al., 2020) and possibly through ENPP2 from hepatocytes (Kaffe et al., 2017), we analyzed the role of activated HSC (aHSC) in HBV-related HCC progression. These data can improve our understanding of the molecular mechanisms in HBV-related HCC malignancy.

## Materials and Methods

### Hepatocellular Carcinoma Patients and Tumor Tissues

The clinicopathologic information of HCC patients and tumor tissues (n = 158) was obtained from Sun Yat-sen Memorial Hospital, Sun Yat-sen University. Patient informed consent was obtained and the procedure of human sample collection was approved by the Ethics Committee of Sun Yat-sen Memorial Hospital (ethical number 202101). Data including primary HCC and adjacent noncancerous liver tissues were obtained from Oncomine (https://www.oncomine.org) and GEPIA (http://gepia.cancer-pku.cn) databases. OS was defined as the interval between the date of surgery and the date of either death or the last follow-up. Disease-free survival (DFS) was defined as the interval between the date of surgery and the date of either tumor recurrence or metastasis.

### Reagents

HBV replication-competent clone pSM2-HBV harboring a head-to-tail tandem dimer of the HBV genome (GenBank accession number: V01460) was provided by Dr. Hans Will (Heinrich-Pette-Institute, Germany). Small interfering RNAs (siRNAs) targeting ENPP2 were synthesized by Genepharma, Shanghai (sequences are listed in Supplementary Table S1). Secreted HBV surface antigen (HBsAg) and HBV e antigen (HBeAg) from cell culture media were detected by Roche Diagnostics (36461400, 33448500, respectively). Cultrex pathclear basement membrane extract (3432-010-01), an ELISA kit for detection of secreted ENPP2 (DENP20), and ENPP2 inhibitor PF-8380 (4078) were purchased from R&D system, United States. Cell counting kit-8 (CCK-8, 40203ES80) was purchased from Yeasen, China.

### Cell Culture and Transfection

Human hepatoma cell lines HepG2, Huh7, immortalized hepatocyte L02, and immortalized activated hepatic stellate cell line LX-2 were maintained in DMEM supplemented with 1% penicillin/streptomycin, 10% fetal bovine serum (FBS, Gibco), and maintained at 37°C in a humidified 5% CO_2_ atmosphere. HepG2.2.15 was cultured with an additional 500 μg/ml of G418 (Apexbio, United States). Plasmid pSM2-HBV (1.5 μg) and siRNAs (30 nM) were transfected into cells seeded in 6-well culture plates using lipofectamine 3000 (Invitrogen, United States) according to the manufacturer’s instructions. The plasmid transfection and HBV expression efficiency were assessed according to published protocols (Deng et al., 2017).

### Western Blotting Analysis

Whole cells were harvested 72 h after transfection and protein samples were subjected to sodium dodecyl sulfate-polyacrylamide gel electrophoresis (SDS-PAGE) and blotted with primary antibodies recognizing ENPP2 (ab77104, Abcam, British) and β-actin (Cell Signing, United States), respectively. Protein bands were visualized using ECL Plus western blotting detection reagents (Millipore, United States).

### Enzyme-Linked Immunosorbent Assay

Secreted HBsAg and HBeAg in culture media from HCC cells were quantified by a double-antibody sandwich ELISA and compared to the cutoff value (the cutoff values were 0.05 for HBsAg and 1 for HBeAg). As for detection of secreted ENPP2, after balancing all reagents in the kit and samples to room temperature (RT), we added 100 μl of assay diluent RD1-34 to each well and then added 50 μl of standard, control, or sample per well in duplicate. The solutions were incubated for 2 h at RT on a horizontal orbital microplate shaker set at 500 rpm. Each well was aspirated and washed with wash buffer (400 μl) four times. A total of 200 μl of human ENPP2 conjugate was added to each well and incubated for 2 h at RT on the shaker. The solutions were aspirated and washed with wash buffer (400 μl) four times. A total of 200 μl of substrate solution was added to each well and incubated for 30 min at RT without light. Then, 50 μl of stop solution was added to each well and the optical density at 450 nm was detected with correction at 540 nm. A standard curve was constructed by plotting the mean absorbance for each standard on the y-axis against the concentration on the x-axis, and the concentration of ENPP2 for each sample was calculated according to the standard curve.

### Quantitative Real-Time Polymerase Chain Reaction

Total RNAs were extracted using TRIzol reagent (Invitrogen, United States) and were reverse-transcribed with a PrimeScriptTM RT Reagent kit (Takara, Japan). RT-qPCR was performed with a 7500 Real-Time PCR system (Thermo Scientific, United States) using TB Green® Premix Ex Taq™ II as described by the manufacturer’s protocol (Takara, Japan). The *enpp2* mRNA expression level was normalized to *gapdh* and quantified by the comparative CT (2^−ΔΔCT^) method and then multiplied by 10^6^. All primers used for RT-qPCR are listed in Supplementary Table S1.

### Cell Viability and Proliferation Assay

Cells were seeded in 96-well culture plates (Corning, United States) at a density of 5,000-10,000 cells/well, each sample seeded two wells. In order to detect the inhibitory ability of PF-8380, hepatoma cells were exposed to PF-8380 on the following day at various concentrations for 48 h. Cell proliferation was then measured by the CCK-8 assay according to the manufacturer’s instructions. The absorbance was determined at 450 nm with a SPARK 10M Microplate Reader (Tecan, Switzerland). The percentage of cell growth inhibition was calculated and the concentration of PF-8380 resulting in a 50% reduction in cell viability was denoted as the 50% inhibitory concentration (IC_50_).

### Cell Migration Assay

The Transwell insert chambers (Corning, United States) was used to assess tumor cell migration ability. Briefly, after having assessed the cell viability, approximately 5–10×10^4^ cells were suspended in serum-free DMEM and seeded into each well of the upper chamber for migration, and DMEM with 20% FBS or conditional media (CM) from LX-2 was added to the lower chamber as a chemoattractant. After 24/48 h of incubation, cells remaining in the upper chamber were removed with cotton swabs. The cells that passed through the membrane were fixed in 4% formaldehyde and stained with 0.1% crystal violet. Cells in at least three random microscopic fields (magnification, ×100) were counted.

### Colony Formation Assay

For the colony formation assay, hepatoma cells were seeded in 6-well culture plates (500-1,000 cells/well), the culture medium or the LX-2 CM was replaced every 48 h. After 2 weeks of incubation, the number of colonies were counted in each well. A colony was counted as such when it had more than 50 cells. The capability of colony formation was evaluated by the colony formation number.

### Immunohistochemistry

The expression of ENPP2 in the paraffin-embedded HCC samples was examined by IHC analysis. IHC experiments were performed as described previously (Xu et al., 2020), and sections were incubated with ENPP2 antibody (ab137590, Abcam, British, 1:400 dilution) overnight at 4°C. To evaluate ENPP2 expression, the scores were determined by the staining intensity and the percentage of positively stained areas. The final staining index was the sum of the staining intensity and extent scores. Staining intensity was scored according to the following standard: zero (negative), one (weak), two (moderate), or three (strong). Scores zero and one and scores two and three were divided into low and high ENPP2 expression groups, respectively. In addition, the extent of staining was scored as follows: zero (0%), one (<10%), two (10–35%), three (36–75%), or four (>75%). Scores zero, one, and two and scores three and four were divided into low and high ENPP2 expression groups, respectively. The clinical samples were reviewed and scored separately by two experienced pathologists.

### *In vivo* Tumor Xenograft Formation

Four-week-old male BALB/c nude mice were purchased from Slyke, Shanghai, China and acclimated to their surroundings for approximately 1 week with food and water before experiments. Animal studies were carried out in the South China University of Technology and approved by the Laboratory Animal Welfare and Ethics Committee of the South China University of Technology (ethical number 2021046). All animal experiments conformed to the approved guidelines of Animal Care and Use Committee of South China University of Technology. All efforts were made to minimize suffering. Mice were randomly divided into six groups (six mice per group): HepG2, LX-2, HepG2+LX-2, HepG2.2.15, HepG2.2.15+LX-2+DMSO, and HepG2.2.15+LX-2+PF-8380. Cells suspended in PBS were mixed with Matrigel with a ratio of 1:1 on ice, and then 200 μl of the cell mixture was seeded into the right flank *via* subcutaneous injection (5×10^6^ cells/mouse and hepatoma cell: LX-2 = 3 : 1). Once tumors were palpable, HepG2.2.15+LX-2 groups were injected intraperitoneally with vehicle (DMSO) or PF-8380 at 30 mg/kg body weight twice daily for 2 weeks. The length and the width of tumors were measured every 3 days. Tumor volume was calculated using the formula (volume = 0.5 × length × width^2^). Mice were anesthetized with isoflurane gas, and sacrificed by cervical dislocation. Tumors were excised, weighed, and their volumes were measured.

### Statistical Analysis

All data are shown as mean ± standard deviation (SD). Statistical significance of differences was detected by Student’s two-tailed *t*-test between two groups and the ANOVA test for multiple comparisons using GraphPad Prism 5 software. Log-rank tests of Kaplan-Meier survival curves were conducted to elucidate the relationship between gene expression and patient survival. Differences were considered statistically significant at *p* < 0.05 (*p < 0.05, **p < 0.01, and ***p < 0.001).

## Results

### Ectonucleotide Pyrophosphatase-Phosphodiesterase 2 Expression Is Positively Correlated to Poor Prognosis in Hepatocellular Carcinoma Patients

**FIGURE 1:**
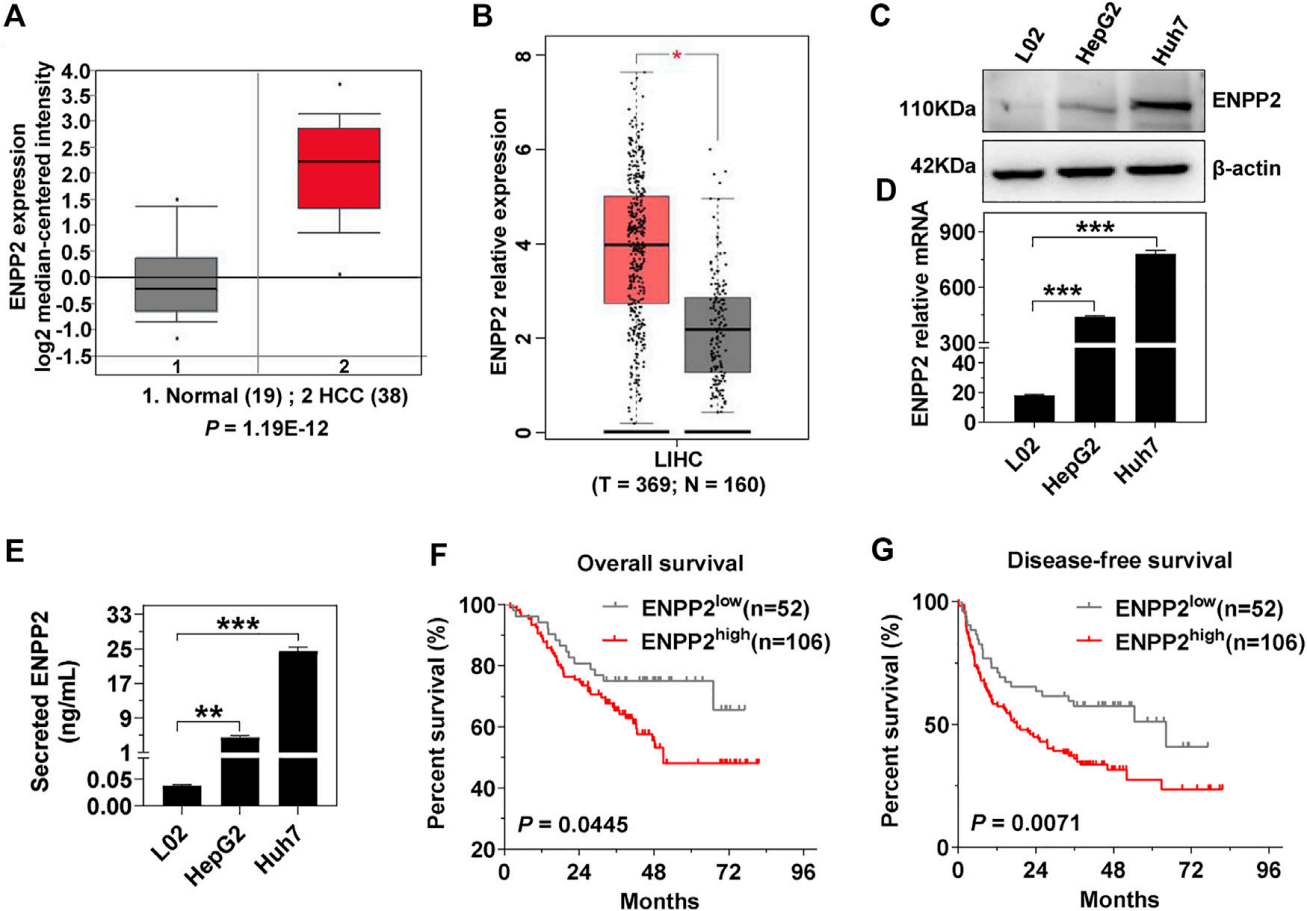
ENPP2 expression is positively correlated with poor patient prognosis. **(A)** Oncomine database analysis of the ENPP2 median-centered intensity in normal (n = 19) and HCC tissue (n = 38). **(B)** GEPIA database analysis of the relative mRNA expression of enpp2 in matched HCC tissues (n = 369) and adjacent normal tissues (n = 160). **(C)** The intracellular ENPP2 protein expression in L02, HepG2, and Huh7, respectively. **(D)** The level of enpp2 mRNA in L02, HepG2, and Huh7, respectively. **(E)** The secreted ENPP2 in supernatants from L02, HepG2, and Huh7, respectively. **(F, G)** Overall survival (F) and disease-free survival (G) for ENPP2^high^ (n = 106) and ENPP2^low^ (n = 52) expression patients, respectively. The statistical analysis was based on three independent experiments. *, *p* < 0.05; **, *p* < 0.01; ***, *p* < 0.001.

Oncomine and GEPIA databases indicated that ENPP2 expression was higher in HCC compared within normal liver tissues (*p* < 0.05; Figures 1A,B). Consistent with clinical data, ENPP2 expression and secretion were significant higher in hepatoma cells, including Huh7 and HepG2, compared within normal hepatocytes both in RNA and protein level (Figures 1C,E). Then liver tissues were collected for IHC from 158 HCC patients who underwent surgery, and the samples were classified into the ENPP2 high expression group (ENPP2^high^, n = 106) and ENPP2 low expression group (ENPP2^low^, n = 52). The OS and DFS were both worse in ENPP2^high^ than ENPP2^low^ (*p* = 0.0445 for OS, *p* = 0.0071 for DFS; Figures 1F,G).

### Hepatitis B Virus Enhances Ectonucleotide Pyrophosphatase-Phosphodiesterase 2 Expression and Secretion

**FIGURE 2:**
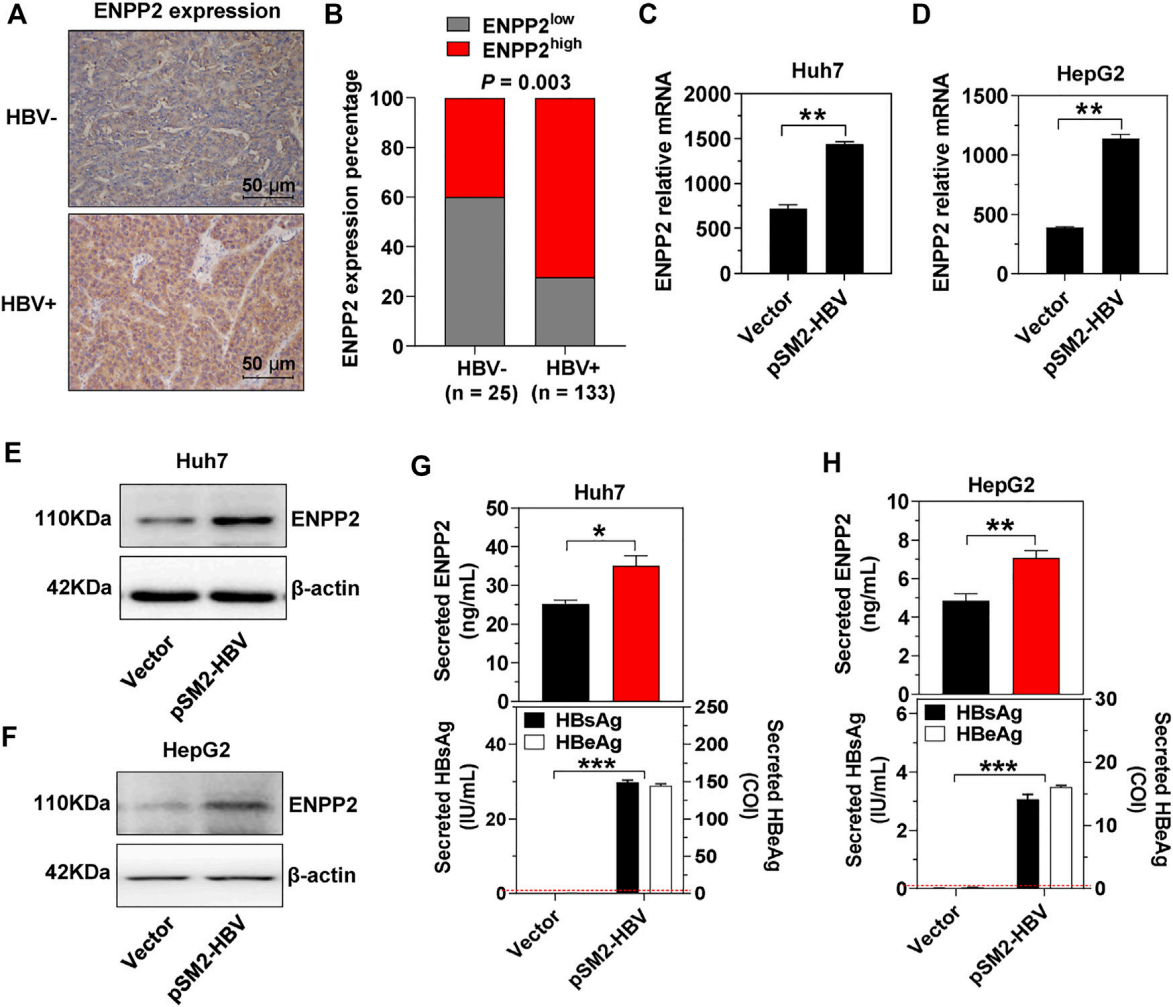
HBV enhances ENPP2 expression and secretion. **(A)** Representative IHC images of ENPP2 staining in HBV- and HBV + HCC tissues (magnification ×200). **(B)** ENPP2 expression percentage in HBV- (n = 25) and HBV + HCC tissues (n = 133), respectively. **(C, D)** The level of enpp2 mRNA in Huh7 (C) and HepG2 (D) after being transfected with vector or pSM2-HBV for 3 days. **(E, F)** The intracellular ENPP2 protein in Huh7 (E) and HepG2 (F) after being transfected with vector or pSM2-HBV for 3 days. **(G, H)** The secreted ENPP2, HBsAg, and HBeAg in supernatants from Huh7 (G) and HepG2 (H) after being transfected with vector or pSM2-HBV for 3 days, respectively. The red dotted line in the graph represents the cutoff for HBsAg and HBeAg. The statistical analysis was based on three independent experiments. *, *p* < 0.05; **, *p* < 0.01.

In the above 158 HCC patients, 25 patients were HBV-negative (HBV-, both HBV DNA and HBsAg were negative in patient serum) and 133 patients were HBV-positive (HBV+, HBV DNA load was more than 5 × 10^2^, and HBsAg was positive in patient serum). In the HBV + cohort, 72.2% of patients had high ENPP2 expression, while only 40.0% of patients displayed high ENPP2 expression in the HBV- cohort, the expression difference between the two cohorts was significant (*p* = 0.003; Figures 2A,B). In order to illuminate the correlation between HBV infection and ENPP2 expression, plasmid pSM2-HBV was transfected into hepatoma cells, then ENPP2 expression and secretion was tested. RT-qPCR results showed that enpp2 mRNA was enhanced by HBV transfection both in Huh7 and HepG2 (Figures 2C,D); western blot and ELISA assays displayed that the intracellular and secreted ENPP2 were also upregulated significantly in the HBV transfected group compared to the control group transfected with the empty vector (Figures 2E–H).

### Hepatitis B Virus Induces Hepatoma Cell Proliferation, Colony Formation, and Migration *via* Upregulation of Ectonucleotide Pyrophosphatase-Phosphodiesterase 2

**FIGURE 3:**
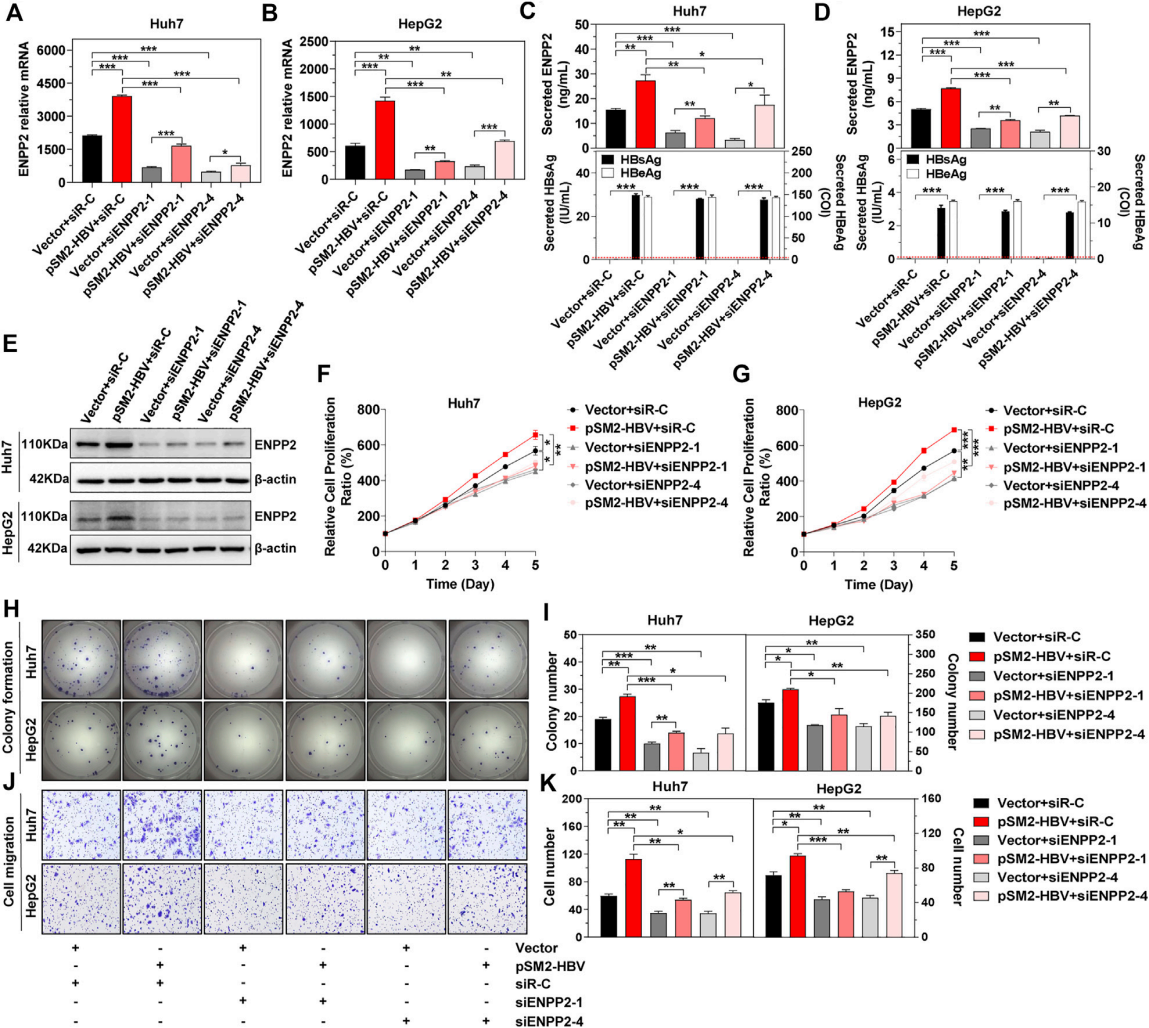
HBV enhances hepatoma cell proliferation, colony formation, and migration, and was impeded by knocking down of ENPP2 expression. Hepatoma cells were firstly transfected with vector or pSM2-HBV for 1 day (set as day 0), and then transfected with siR-C or siENPP2. **(A, B)** The level of enpp2 mRNA in Huh7 (A) and HepG2 (B) after being transfected with siRNAs for 3 days. **(C, D)** Secreted ENPP2, HBsAg, and HBeAg in supernatants from Huh7 (C) and HepG2 (D) after being transfected with siRNAs for 3 days. The red dotted line in the graph represents the cutoff for HBsAg and HBeAg. **(E)** The intracellular ENPP2 expression in Huh7 **(upper panels)** and HepG2 **(below panels)** after being transfected with siRNAs for 3 days. **(F, G)** The cell proliferation ability for Huh7 (F) and HepG2 (G), respectively. **(H)** Representative images of colony formation for Huh7 **(upper panels)** and HepG2 **(below panels)**, respectively. **(I)** The bar graph of colony number indicated quantitative analysis data for Huh7 **(left panel)** and HepG2 **(right panel)**, respectively. **(J)** Representative images of the cell migration assay for Huh7 **(upper panels)** and HepG2 **(below panels)**, respectively (magnification ×100). **(K)** The bar graph of cell number migrated from Transwell indicated quantitative analysis data for Huh7 **(left panel)** and HepG2 **(right panel)**, respectively. The statistical analysis was based on three replicates. *, *p* < 0.05; **, *p* < 0.01; ***, *p* < 0.001.

Next, in order to elucidate whether HBV induces HCC progression *via* regulation of ENPP2, plasmid pSM2-HBV was transfected into hepatoma cells to express the HBV genome, then cells were re-transfected with siRNAs to knock down ENPP2 expression. The knockdown efficiencies for the two selected siRNAs targeting ENPP2 (siENPP2-1 and siENPP2-4) were more than 70% (Supplementary Figure S1). The immunoblotting results confirmed that HBV transfection enhanced ENPP2 expression and secretion both at the RNA and protein level, while knocking down of ENPP2 weakened these phenomena both in Huh7 and HepG2 (Figures 3A–E). The CCK-8 assay displayed that compared to those transfected with control vector, hepatoma cells transfected with pSM2-HBV had a stronger ability of proliferation, while knocking down of ENPP2 could inhibit cell proliferation significantly (Figures 3F,G). The colony formation assay confirmed that HBV enhanced hepatoma cell proliferation and this phenomenon could be weakened by knocking down ENPP2 expression both in Huh7 and HepG2 (Figures 3H,I). HBV also promoted hepatoma cell migration, while knocking down of ENPP2 could inhibit hepatoma cell migration even though cells were transfected with pSM2-HBV (Figures 3J,K).

**FIGURE 4:**
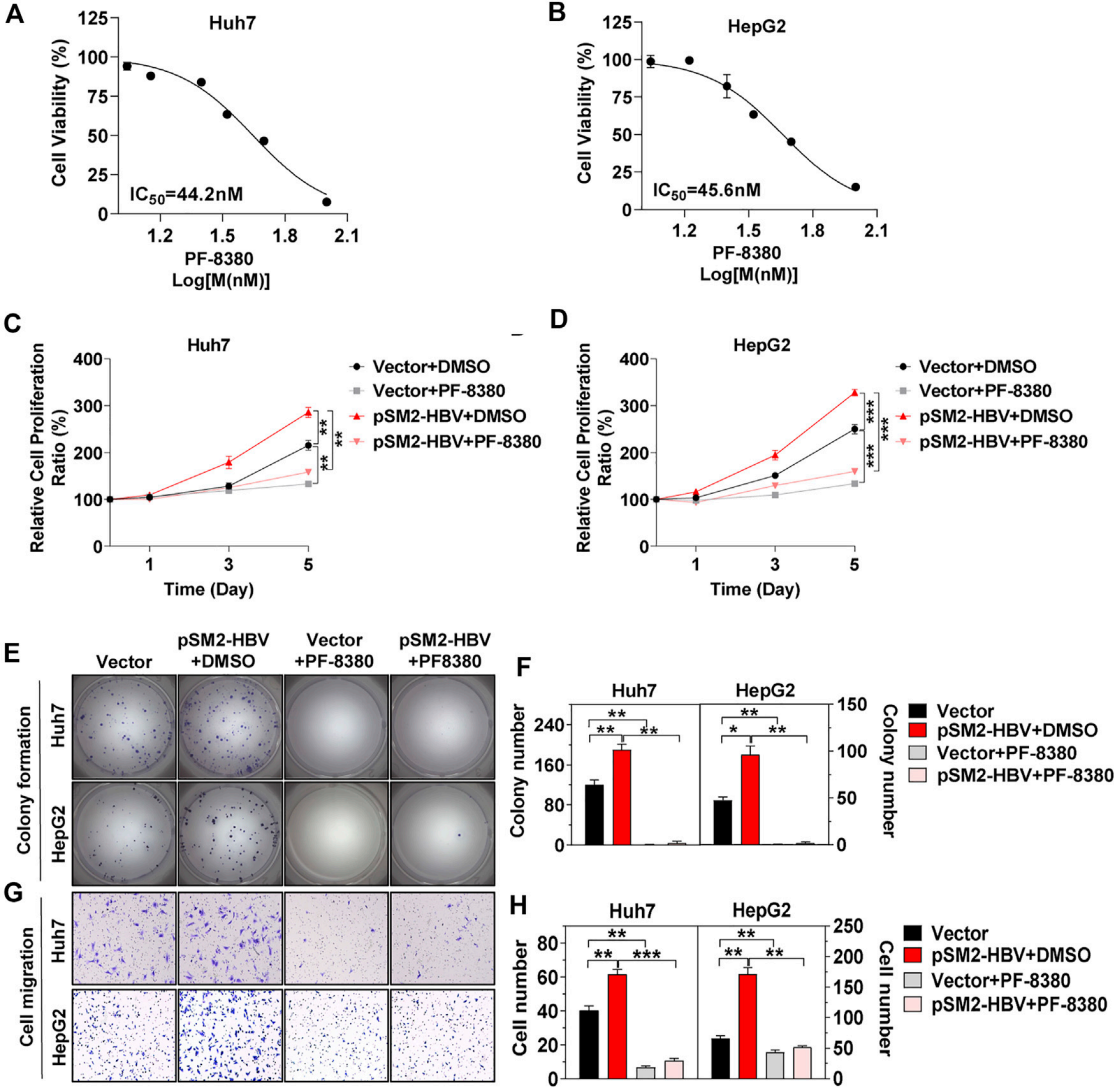
HCC progression induced by HBV is impeded by inhibitor PF-8380. Hepatoma cells were transfected with vector or pSM2-HBV for 1 day (set as day 0) and then treated cells with vehicle DMSO or ENPP2 inhibitor PF-8380. **(A, B)** Antiproliferative activity of PF-8380 against Huh7 (A) and HepG2 (B) *in vitro.* **(C, D)** A CCK-8 assay was carried out to detect cell proliferation for Huh7 (C) and HepG2 (D), respectively. **(E)** Representative images of colony formation for Huh7 **(upper panels)** and HepG2 **(below panels)**, respectively. **(F)** The bar graph of colony number indicated quantitative analysis data for Huh7 **(left panel)** and HepG2 **(right panel)**, respectively. **(G)** Representative images of the cell migration assay for Huh7 **(upper panels)** and HepG2 **(below panels)**, respectively (magnification ×100). **(H)** The bar graph of cell number migrated from Transwell indicated quantitative analysis data for Huh7 **(left panel)** and HepG2 **(right panel)**, respectively. The statistical analysis was based on three replicates. *, *p* < 0.05; **, *p* < 0.01; ***, *p* < 0.001.

Then PF-8380, a small molecule inhibitor that suppresses the activity of ENPP2 and has a reported IC_50_ of 1.7 nM on natural LPC substrates, was used (Gierse et al., 2010). *In vitro,* the inhibition on hepatoma cell viability was tested, and the IC_50_ on Huh7 and HepG2 were 44.2 and 45.6 nM, respectively (Figures 4A,B). PF-8380 inhibited Huh7 and HepG2 proliferation obviously even though the hepatoma cells were under the condition of HBV transfection (Figures 4C,D). The colony formation assay confirmed that HBV was enhanced, but PF-8380 inhibited hepatoma cell colony formation obviously (Figures 4E,F). The cell migration assay showed that PF-8380 impeded the ability of migration for Huh7 and HepG2 even though the cells were transfected with pSM2-HBV (Figures 4G,H).

### Hepatocellular Carcinoma Progression Induced by Hepatitis B Virus Is Strengthened by Activated Hepatic Stellate Cells

**FIGURE 5:**
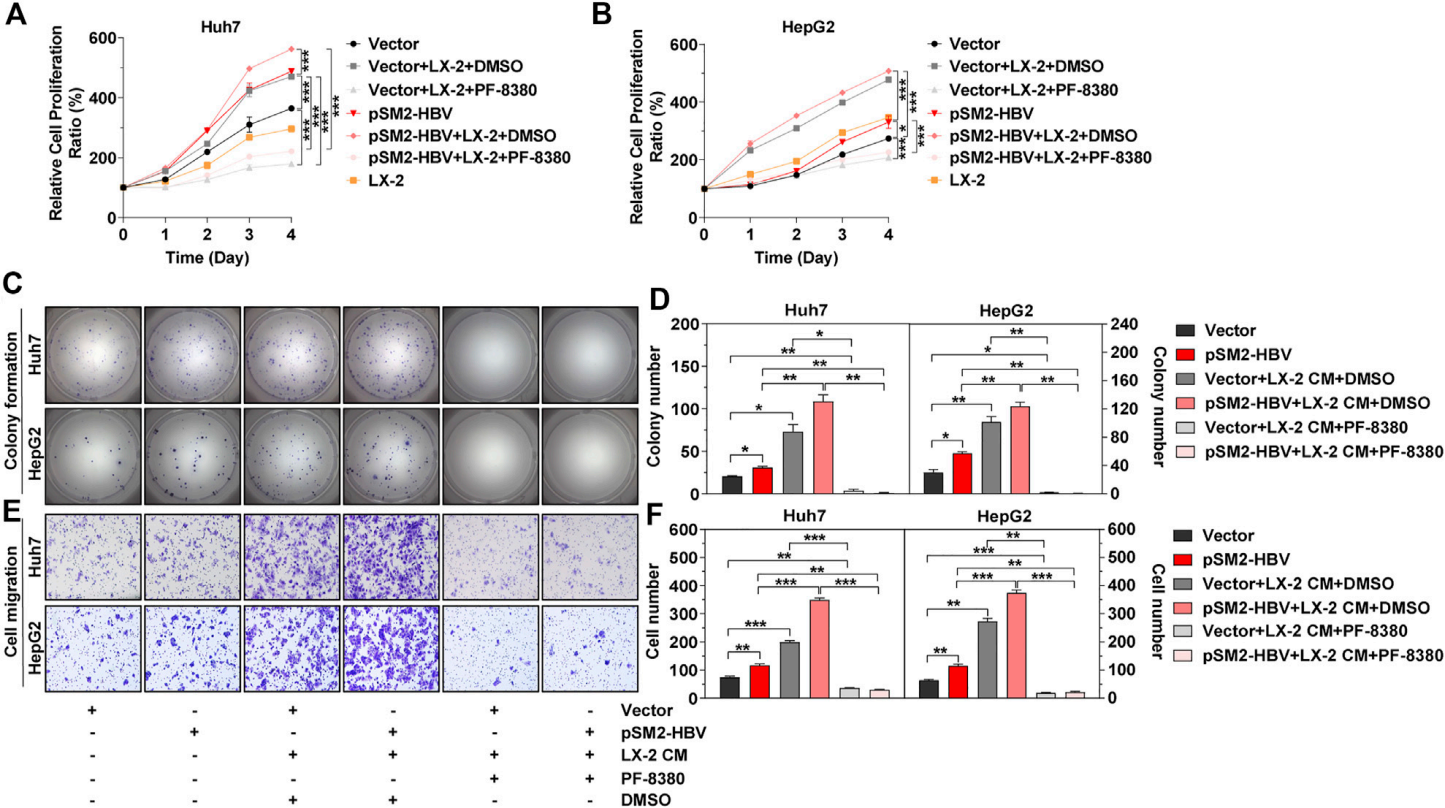
Activated HSC strengthens HCC progression induced by HBV. Hepatoma cells were transfected with vector or pSM2-HBV for 1 day (set as day 0) and then co-cultured with LX-2 or treated with LX-2 CM containing DMSO or PF-8380. **(A, B)** Cell proliferation abilities for Huh7 (A) or HepG2 (B), respectively. **(C)** Representative images of colony formation for Huh7 **(upper panels)** and HepG2 **(below panels)**, respectively. **(D)** The bar graph of colony number indicated quantitative analysis data for Huh7 **(left panel)** and HepG2 **(right panel)**, respectively. **(E)** Representative images of the cell migration assay for Huh7 **(upper panels)** and HepG2 **(below panels)**, respectively (magnification ×100). **(F)** The bar graph of cell number migrated from Transwell indicated quantitative analysis data for Huh7 **(left panel)** and HepG2 **(right panel)**, respectively. The statistical analysis was based on three replicates. *, *p* < 0.05; **, *p* < 0.01; ***, *p* < 0.001.

The above results confirmed that HBV promoted tumor cell proliferation and migration *via* enhancement of ENPP2 expression and secretion in hepatoma cells. A major contributor to tumor progression is the cross talk between tumor cells and the surrounding stroma. HSC is the major surrounding cell in the liver tumor microenvironment and aHSC normally promotes HCC progression (Barry et al., 2020; Ruan et al., 2020). So co-culture assays were performed between hepatoma cells and aHSC, the effects of these interactions were then studied by a series of functional assays. The CCK-8 assay showed that cell proliferation was enhanced obviously when co-culturing HepG2 or Huh7 with LX-2 compared to cultured hepatoma cells alone, especially when hepatoma cells were transfected with pSM2-HBV (Figures 5A,B). While the inhibitor PF-8380 could suppress cell proliferation even though the hepatoma cells were both under pSM2-HBV transfection and co-cultured with LX-2 (Figures 5A,B). Hepatoma cells were also incubated with LX-2 CM and the results showed that LX-2 CM significantly enhanced the colony formation for Huh7 and HepG2, especially when hepatoma cells were transfected with pSM2-HBV, while these phenomena were suppressed by PF-8380 (Figures 5C,D). In addition to cell proliferation, LX-2 CM also enhanced Huh7 and HepG2 migration, especially when hepatoma cells were transfected with pSM2-HBV, while these phenomena were also suppressed by PF-8380 (Figures 5E,F).

### Hepatitis B Virus and Activated Hepatic Stellate Cells Induce Hepatocellular Carcinoma Progression Synergistically *in vivo*

**FIGURE 6:**
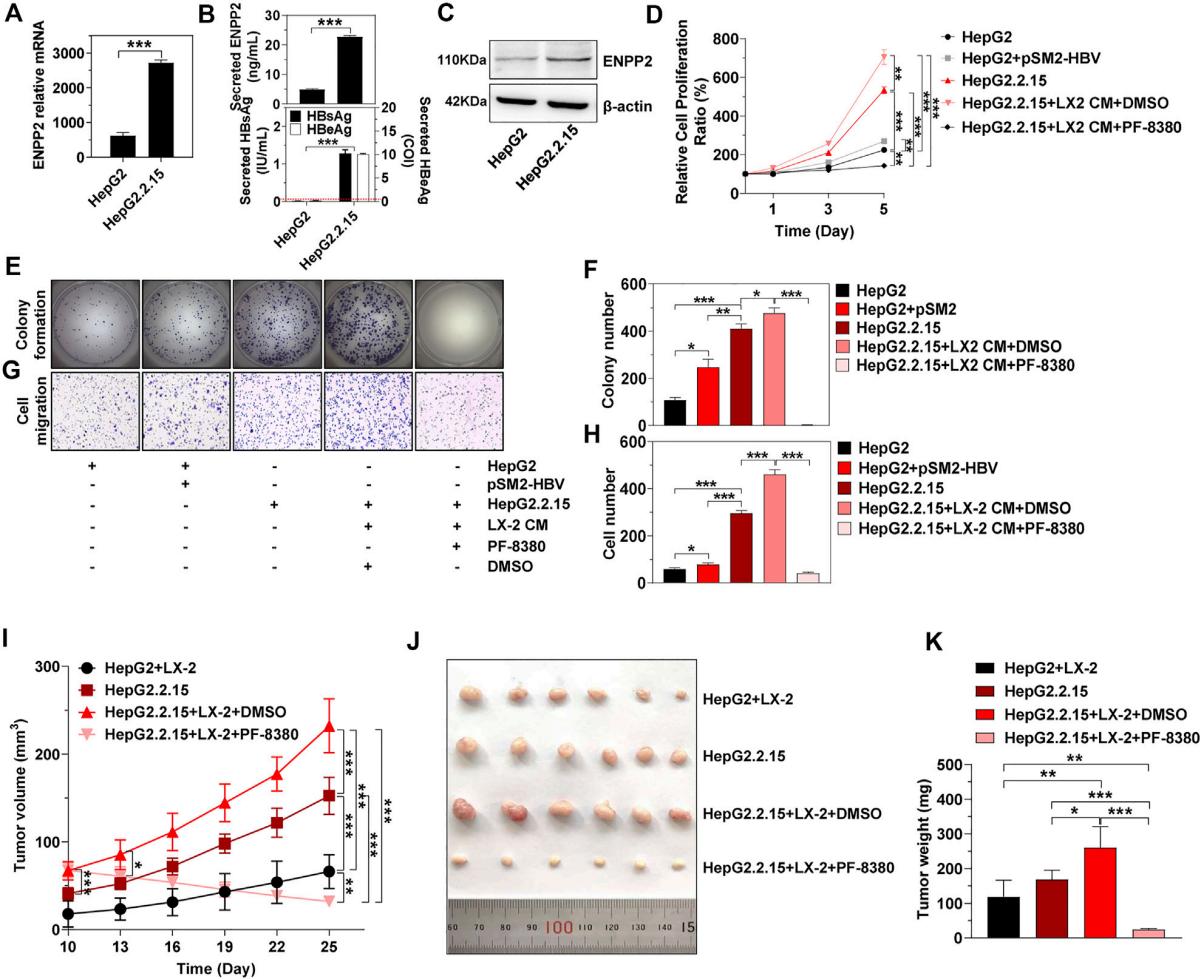
HBV and aHSC induce HCC progression synergistically *in vivo*. **(A)** The level of enpp2 mRNA in HepG2 and HepG2.2.15. **(B)** The secretory ENPP2, HBsAg, and HBeAg in supernatants from HepG2 and HepG2.2.15. The red dotted line in the graph represented the cutoff for HBsAg and HBeAg. The statistical analysis was based on three independent experiments. **(C)** The intracellular ENPP2 protein in HepG2 and HepG2.2.15. HepG2.2.15 and HepG2, transfected with vector or pSM2-HBV for 1 day (set as day 0), were treated with LX-2 CM containing DMSO or PF-8380, and then hepatoma cell function was detected. **(D)** Cell proliferation for each group. The statistical analysis was based on three replicates. **(E)** Representative images of colony formation for each group cells. (**F**) The bar graph of colony number indicated quantitative analysis data based on three independent experiments for each group of cells. **(G)** Representative images of the cell migration assay for each group of cells (magnification ×100). **(H)** The bar graph of cell number migrated from Transwell indicated quantitative analysis data based on three independent experiments for each group of cells. **(I)** Tumor growth at different times in each group in nude mice (error bars, SD of six mice per group). **(J)** The final tumor size in nude mice upon subcutaneous transplantation with hepatoma cells or co-transplantation with hepatoma cells with LX-2 (3:1 ratio). Indicated groups were treated with vehicle DMSO or PF-8380 for two consecutive weeks. **(K)** Bar charts show the final tumor weight of each group of mice. *, *p* < 0.05; **, *p* < 0.01; ***, *p* < 0.001.

To explore whether HBV determined the tumorigenicity of HCC *in vivo*, we used HepG2.2.15, a cell line derived from HepG2, to integrate and express the HBV whole genome stably. Compared to HepG2, there was much more intracellular and secretory ENPP2 in HepG2.2.15 (Figures 6A–C). The proliferation, colony formation, and migration ability of HepG2.2.15 were stronger than HepG2 or HepG2 transfected with pSM2-HBV, especially when HepG2.2.15 was incubated with LX-2 CM, while PF-8380 could inhibit HepG2.2.15 proliferation and migration even though the cells were incubated with LX-2 CM (Figures 6D–H).

Then subcutaneous transplantation of HepG2 and HepG2.2.15, or co-transplantation of HepG2 and HepG2.2.15 with LX-2 into immune-compromised nude mice was carried out to observe tumor growth. There was no tumor formation in mice injected either with HepG2 or LX-2 alone, while mice co-injected with HepG2 and LX-2 (HepG2+LX-2) had obvious tumor formations on day 10 (Figure 6I). Mice injected with HepG2.2.15 alone also formed tumors, the tumors in mice co-injected with HepG2.2.15 and LX-2 (HepG2.2.15+LX-2) were larger than those injected with HepG2.2.15 alone on day 10 (*p* < 0.001) (Figure 6I). To further assess that ENPP2 was the key factor in liver tumor formation in this mouse model, intraperitoneal injection of PF-8380 or DMSO was conducted in mice subcutaneously transplanted with HepG2.2.15+LX-2. Compared to DMSO, intraperitoneal injection of PF-8380 significantly suppressed tumor growth (*p* < 0.05 on day-13), and after injection of an inhibitor for two consecutive weeks, the tumors in the HepG2.2.15+LX-2+PF-8380 group were almost undetectable. While the average size of tumors in the HepG2.2.15+LX-2+DMSO group was 232 mm^3^ **(** *p* < 0.001), other groups, including the HepG2+LX-2 and HepG2.2.15 groups also had growing tumors, and the volume of tumors among these four groups had significant differences on day 25 (Figures 6I,J). The tumor weight among these four groups also had statistic differences except between HepG2+LX-2 and HepG2.2.15 groups (Figure 6K).

## Discussion

Differences in the etiologies and pathogenic mechanisms of hepatocarcinogenesis may reflect the unique clinical characteristics and prognosis in patients with HCC, and patients with negative HBV infection normally have better prognosis and lower risk of tumor recurrence than HBV-positive patients (Utsunomiya et al., 2015; Liu A. P. Y. et al., 2020). In the present study, our *in vitro* and *in vivo* results verified that transient transfection or stable expression of the HBV genome enhanced hepatoma cell proliferation and migration and also promoted xenograft tumor formation in nude mice. These mean antiviral treatments still act as key approaches to improve patient prognosis and could significantly decrease tumor recurrence and tumor-related death for HBV-related HCC patients.

Increased ENPP2 expression is detected in chronic liver disease patients of different etiologies, including HBV-associated liver disease (Kaffe et al., 2017; She et al., 2018). The data from cancer databases confirmed that ENPP2 was highly expressed in HCC tissue compared within normal liver tissues, our clinical data displayed that HCC patients with high ENPP2 level normally had poor prognosis, and ENPP2 expression was positively correlated with HBV infection. Besides, our results indicated that knocking down of ENPP2 expression or inhibiting ENPP2 function could impede hepatoma cell proliferation, migration, and xenograft tumor formation induced by HBV. Mechanistically, ENPP2 hydrolyzes LPC to produce LPA (Salgado-Polo et al., 2018), a lipid mediator that functions as a mitogen and motility factor to stimulate proliferation, migration, and survival of cancer cells through combination with the LPA receptors (LPAR) (Houben and Moolenaar, 2011), which are overlapping specificities and have widespread distribution (Choi et al., 2010). Bioinformatics analysis suggests that lipid metabolisms are strongly activated by HBV-associated proteins and lead to the progression of liver tumors (Xu et al., 2016). These indicate that ENPP2 induced by HBV may promote HCC progression *via* downstream LPA/LPAR in HBV-related HCC patients.

Besides altering gene expressions in host cells, virus infection often transfers environment. It is reported that HBV infection causes liver fibrosis *via* activation of HSC (Zan et al., 2013; Gong et al., 2016; Zhang et al., 2020). In our results, we confirmed that HBV enhanced the secretion of ENPP2 in hepatoma cells, and ENPP2 is reported to have the function to activate HSC (Kaffe et al., 2017), these points imply that HBV may activate HSC *via* ENPP2. On the other hand, approximately 90% of HCC cases arise in the context of liver fibrosis (Seitz and Stickel, 2006), during the hepatic fibrotic process, activation of HSC drives fibrogenesis, changes the composition of the extracellular matrix, and is considered to be the central event that contributes to hepatic malignancies (Santamato et al., 2011; Zhang and Friedman, 2012; Coulouarn and Clement, 2014). Our co-culture assays showed that aHSC could strengthen hepatoma cell proliferation, colony formation, migration, and tumor formation induced by HBV *via* ENPP2 both *in vitro* and *in vivo*. In pancreatic ductal adenocarcinoma (PDAC), LPCs secreted from activated pancreatic stellate cells could be hydrolyzed by ENPP2, which was produced by PDAC, and finally promoted pancreatic tumor progression *via* the ENPP2/LPA axis (Auciello et al., 2019; Biffi and Tuveson, 2019), so in future, we need to clarify if aHSC may also secrete LPCs acting as a major substrate for ENPP2, and if HBV induces HCC progression not only by autocrine ENPP2, but also transfers the microenvironment to benefit HCC survival *via* the LPC/ENPP2/LPA axis in HBV-related HCC patients.

In summary, this study clarified that HBV infection induced ENPP2 expression and secretion in hepatocytes. HSC could synergistically enhance hepatoma cell proliferation, migration, and liver tumor formation with HBV *via* ENPP2. These indicate that besides antiviral therapy, ENPP2 could also be considered as a key regulation factor in improving prognosis for HBV-related HCC patients.

## Data Availability

The original contributions presented in the study are included in the article/Supplementary Material, further inquiries can be directed to the corresponding authors.
