# Supplementary material for: Hepatitis B Virus Promotes Hepatocellular Carcinoma Progression Synergistically With Hepatic Stellate Cells via Facilitating the Expression and Secretion of ENPP2
Source: Front Mol Biosci. 2021 Nov 5;8:745990. doi: 10.3389/fmolb.2021.745990 (PMC8602366; doi:10.3389/fmolb.2021.745990)
Supplement: Supplementary file 1 [file DataSheet1.doc]

Supplementary Material

# Supplementary Tables

**Supplementary Table 1: The sequences of siRNAs and primers**

| **Name** | **Sequences 5’-3’** |
| --- | --- |
| siENPP2-1-sense | GGAAAUAAAGGCCGCAGAATT |
| siENPP2-1-antisense | UUCUGCGGCCUUUAUUUCCTT |
| siENPP2-2-sense | GGACACAAAUAUGGCCCUUTT |
| siENPP2-2-antisense | AAGGGCCAUAUUUGUGUCCTT |
| siENPP2-3-sense | CCAAUUAUCCAGGGAUUAUTT |
| siENPP2-3-antisense | AUAAUCCCUGGAUAAUUGGTT |
| siENPP2-4-sense | GCUCUUCACCAGAGGCUAATT |
| siENPP2-4-antisense | UUAGCCUCUGGUGAAGAGCTT |
| siRNA-negative control-sense | UUCUCCGAACGUGUCACGUTT |
| siRNA-negative control-antisense | ACGUGACACCGUUCGGAGAATT |
| hsa-GAPDH forward | CGCTGAGTACGTCGTGGAGTC |
| hsa-GAPDH reverse | GCTGATGATCTTGAGGCTGTTGTC |
| hsa-ENPP2 forward | AACTCGGAGAAACACGGACAT |
| hsa-ENPP2 reverse | ACGGCTTCATACAAAAGGGTC |

The human siRNAs were provided by GenePharma (Shanghai, China). The primers for RT-qPCR were synthesized by Generay (Shanghai, China).

**Supplementary Figures**

**A**

**B**

**Supplementary Figure 1** | ENPP2 knocking down efficiency by siRNAs in hepatoma cells. (**A**, **B)** Four siRNAs targeting ENPP2 were transfected into Huh7 (A) or HepG2 (B) for three days, then detection *ENPP2* mRNA then detection *ENPP2* mRNA by RT-qPCR. The *ENPP2* mRNA expression levels were normalized to those of *GAPDH* and quantified by the comparative CT (2-ΔΔCT) method and then multiplication by 10^6^. All experiments were done in triplicate.
